# Computer Use and Compressive Neuropathies of the Upper Limbs: A Hidden Risk?

**DOI:** 10.3390/jcm14155237

**Published:** 2025-07-24

**Authors:** Georgiana-Anca Vulpoi, Cătălina Elena Bistriceanu, Lenuța Bîrsanu, Codrina-Madalina Palade, Dan Iulian Cuciureanu

**Affiliations:** 1 Neurology Department, Faculty of Medicine, University of Medicine and Pharmacy “Grigore T. Popa”, 16 Universitatii Street, 700115 Iasi, Romaniacuciureanudan@yahoo.com (D.I.C.); 2 Elytis Hospital Hope, 43A Gheorghe Saulescu Street, 700010 Iasi, Romania; 3 Department of Neurology, Rehabilitation Hospital, 700661 Iasi, Romania; 4 “Socola” Institute of Psychiatry, 700282 Iasi, Romania; codrina.palade20@gmail.com; 5 Neurology Department I, “Prof. Dr. N. Oblu” Emergency Clinical Hospital, 2 Ateneului Street, 700309 Iasi, Romania

**Keywords:** peripheral neuropathies, carpal tunnel syndrome, cubital tunnel syndrome, computer use, nerve entrapment, electrophysiological studies

## Abstract

In recent decades, information technology has grown. Computers have become a daily activity, facilitating access to information, faster communication and faster work. If used responsibly, it has many advantages. **Objectives:** To explore the potential link between prolonged use of computer input devices—such as keyboards and mice—and the development of compressive neuropathies, including carpal tunnel syndrome (CTS) and cubital tunnel syndrome (CuTS), in individuals whose daily routines are heavily reliant on computer-based activities. **Methods:** A comprehensive review of the literature was undertaken to assess the correlation between the use of computer input devices and the incidence of compressive neuropathies in the upper limbs, with particular attention to repetitive strain, ergonomic posture deviations, and personal risk factors. **Results:** Current evidence indicates a potential association between prolonged computer use and the development of upper limb compressive neuropathies; however, a definitive consensus within the scientific literature remains elusive. Repetitive movements and non-neutral postures appear to be significant contributing factors, particularly among individuals with predisposing risk factors. Despite increasing awareness of this issue, standardized, evidence-based clinical guidelines for the evaluation and management of work-related nerve disorders remain lacking. **Conclusions:** While the relationship between computer use and compressive neuropathies remains debated, healthcare professionals should be aware of the risks, particularly in individuals exposed to repetitive strain and ergonomic stress. Further research and the development of clinical guidelines are needed to better understand and manage these work-related conditions.

## 1. Introduction

Work-related musculoskeletal disorders (WMSDs) affect an increasing number of office workers, whose symptoms can have a negative impact not only on their work productivity but also on their quality of life [1]. Neck and upper extremity musculoskeletal disorders are associated with computer work [2]. The diagnosis of compression neuropathies of upper limbs is common among working individuals. They generally have an insidious onset, are multifactorial, and a clear causality is lacking [3]. Carpal tunnel syndrome (CTS) has been found to have become more common among office workers, especially computer workers [4].

Median neuropathy at the wrist (MNW) and ulnar neuropathy at the elbow (UNE) are the most common compression neuropathies of the upper limb. CTS is an association of symptoms and signs, assumed to be caused by MNW [5]. An individual without symptoms, such as pain, paresthesia, or loss of skilled hand movement, cannot be classified as having CTS [5]. A prevalence of 4.9% of the general population suffers from symptoms associated with MNW and electrophysiological median neuropathy [6].

The ulnar nerve is the second most affected nerve in the upper limb in terms of susceptibility to compression. Along its anatomical course, it presents several potential sites for entrapment. The most frequent site of compression is at the elbow, specifically within the cubital tunnel. This is followed by compression at the wrist level, occurring within Guyon’s canal [7,8]. Guyon’s canal syndrome is a relatively uncommon form of peripheral ulnar neuropathy, for which precise incidence and prevalence rates in the general population remain undetermined, largely due to the limited availability of epidemiological studies [9]. In the literature, an association is described between CTS and ulnar tunnel syndrome or ulnar neuropathy at the wrist (UNW), as they are anatomically adjacent. The transverse carpal ligament forms the roof of the carpal tunnel and the floor of the ulnar tunnel. This allows pressure from within the carpal tunnel to be transmitted to the ulnar tunnel, and vice versa [9].

**Figure 1:**
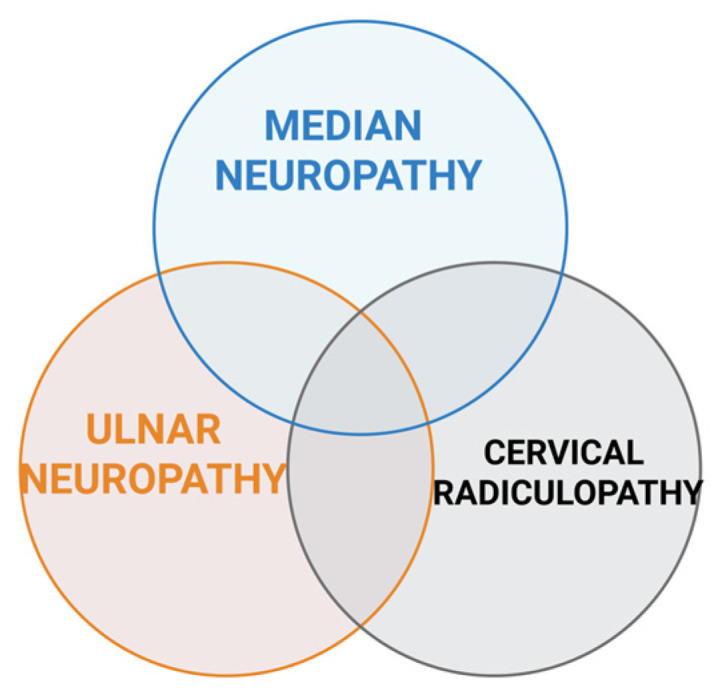
Double crush syndrome affecting the peripheral nerves of the upper limbs (created in BioRender. Vulpoi, A. (2025) https://BioRender.com/fi46ba8, accessed on 17 May 2025).

Prolonged computer use, particularly when combined with awkward sitting posture and repetitive upper limb movements, has been associated with neck pain. Improper posture may increase pressure and intradiscal load on the zygapophyseal joints of the cervical spine, potentially leading to discomfort and pain [10]. There is a clinical condition known as double crush syndrome (DCS), which involves multiple compression sites along a single peripheral nerve. Upper limb involvement is most common, with the proximal lesion on the root or plexus and the distal lesion along one of the major peripheral nerves: median, ulnar, or radial. Among the most thoroughly investigated associations is that between CTS and cervical radiculopathy (Figure 1). Another DCS is UNE associated with UNW [11]. Upton and McComas postulated that compression of one site of the axon may lead to injury in other parts. It is caused by a disruption in bidirectional nutrient transport in the axon [11]. Due to the lack of standardized and validated criteria for defining and diagnosing DCS, there is no real consensus regarding its epidemiology [11].

We live in a digital world, and a question arises as to whether excessive computer usage leads to upper limb compressive neuropathies and if so, should this be considered an occupational hazard [12]. Furthermore, it is worth considering whether individuals at risk would benefit from ongoing monitoring of symptoms, timely and accurate diagnosis, and the implementation of preventive management strategies. At present, there is a lack of evidence-based guidelines to assist clinicians in evaluating the impact of work-related nerve dysfunction.

## 2. Methodology

This review employed a narrative synthesis approach to explore the association between prolonged computer use and compressive neuropathies of the upper limbs. It focused on CTS and CuTS. The methodological objective was to compile, summarize, and critically analyze the current evidence related to the pathophysiology, epidemiology, risk factors, diagnostic considerations and preventions recommendations of these conditions. In this narrative review, the literature search was conducted in two of the most relevant online databases (PubMed and Google Scholar), using keywords related to the topics discussed in the study, published up to May 2025. The following terms were searched: “carpal tunnel syndrome”, “cubital tunnel syndrome”, “median neuropathy”, “ulnar neuropathy”, “computer use”, “mouse”, “keyboard”, “diagnosis”, “prevalence”, “ergonomics”, and “risk factors”. The following terms in the Medical Subject Headings (MeSH) are used to refer to imaging techniques and electrophysiological investigations: “NMUS” for “Neuromuscular Ultrasound”, “NCS” for “Nerve Conduction Studies”, “EMG” for “Electromyography”, “MRI” for “Magnetic Resonance Imaging”.

Only studies that met the following criteria were included: publications in English, human studies involving adult subjects, research articles, systematic reviews, meta-analyses, cross-sectional studies, cohort studies, and case–control designs. Included studies assessing prevalence, pathophysiology, diagnostic approaches, or risk factors of compressive neuropathies associated with computer use. Studies were excluded if they: focused exclusively on pediatric, were published in languages other than English, or consisted of conference abstracts, editorials, and letters without original data. Data was extracted solely from published sources, which were appropriately cited throughout the manuscript.

## 3. Epidemiology of Compressive Neuropathies of the Upper Limb in Computer Users

**Median neuropathy**: The prevalence of CTS varies greatly depending on the diagnostic criteria, population, and type of study and can range from 0.6 to 61% [12,13]. Office work-related CTS prevalence has been reported to range from approximately 5000 to 7500 cases per 100,000 people [14]. There was an association between hand/wrist symptoms and working on a computer for more than five hours and an uncomfortable sitting posture [15,16].

In computer professionals, CTS is a common musculoskeletal disorder. Repetitive hand motions can aggravate a preexistent CTS or determine it, particularly in people whose jobs require strong finger and wrist movements [4]. There were 29.6% of frequent computer users who reported paresthesia and 10.5% who met clinical criteria for CTS in a survey of employees. In 3.5% of cases the syndrome was confirmed by nerve conduction studies (NCS) [17].

Several high-quality systematic reviews and meta-analyses, including those by Thomsen et al. (2008) and Shiri & Falah-Hassani (2015), have been published in recent years, providing comprehensive summaries of the current knowledge on this subject [18,19]. Their findings suggest that the epidemiological evidence linking computer use to CTS remains inconclusive and no definitive studies have yet been found to clarify this relationship. Shiri R et al. conclude, however, that excessive computer use—particularly prolonged mouse use—may be a minor occupational risk factor [18,19].

Also, based on their meta-analysis, Mediouni Z et al. (2014) concluded that there is inconclusive evidence supporting the role of computer use in CTS [20]. There was no association between keyboard use and CTS, but mouse users may be at a higher risk for CTS [20]. On the other hand, Bibi M et al. (2019), based on the included literature, concludes that prolonged use of the keyboard and mouse is one of the main contributing factors to the development of CTS [21]. Table 1 presents an overview of meta-analyses and systematic reviews published to date regarding the potential association between computer use and CTS, along with their respective conclusions.

Due to the lack of clarity in meta-analyses and systematic reviews on this topic, studies have continued to evaluate the link between CTS and computer use and determined the prevalence among computer users. Mediouni Z et al.’s [22] observational longitudinal cohort study found no overall association between computer use and CTS. It does not exclude the possibility that specific biomechanical exposures to certain types of computer work may increase the risk of CTS. This is especially common among workers not engaged in other hand-intensive tasks. As a result of the research, it was concluded that computer use was associated with a reduced risk of CTS when compared with workers in multiple industries [22]. Another study, by Bhanderi D et al. also found a negative association between computer use and CTS. This was consistent with the Atroshi et al. study [23,24]. On the other hand, in their studies, Bibi M et al. concluded that prolonged use of keyboard and mouse are some of the major contributing factors for [25]. Also, Feng B et al. and Demissie B et al. found a relatively high prevalence rate of work-related wrist and hand complaints and clinically confirmed CTS among office workers [1,26]. In a cross-sectional study by Feng B et al., CTS has been clinically confirmed to be a highly prevalent condition among office workers (9.6%). Studies have revealed that prolonged computer use has been associated with wrist and hand pain. The risk of being diagnosed with CTS was 3 to 4 times greater for employees who frequently or always work in pain [1]. Study results by Kurane et al. found that CTS is commonly reported among non-teaching staff who regularly use computers. Age, long-term employment, and prolonged computer use are key risk factors [27]. The number of hours spent in front of these devices correlates with the appearance of WMSDs of the upper extremities [19]. This is associated with absence sickness, decrease in productivity and increased disability payments [28].

Table 2 provides an overview of studies published in the past decade examining the potential association between computer use and CTS, with a focus on prevalence.

The American Conference of Governmental Industrial Hygienists (ACGIH) is a private, non-profit scientific association that publishes Threshold Limit Value (TLV) guidelines for safe levels of exposure to biological, chemical, and physical agents in the workplace [29]. The ACGIH employs a hand-activity level (HAL) assessment tool designed to evaluate the risk of work-related hand and musculoskeletal disorders of workers who engage in repetitive hand motion for 4 or more hours per day. Any activity above TLV puts the worker at risk of developing musculoskeletal disorders [29]. Kozal et al. found a positive correlation between the ACGIH HAL value and the risk of developing CTS [30]. CTS has become a topic of concern not only for healthcare professionals but also for employers, policy makers and occupational health specialists, reflecting its wide-ranging implications. This increasing prevalence highlights the need for an approach that encompasses prevention, early diagnosis and good management [31].

**Ulnar neuropathy**: Few epidemiological studies assessed the prevalence of ulnar neuropathy in the working population. Table 3 presents studies assessing the relationship between computer use and ulnar neuropathy—both at the elbow (UNE) and wrist (UNW)—with a particular focus on prevalence.

UNE has a prevalence of approximately 0.6–0.8%, found in a French working population, reaching over 6% in self-reported questionnaires [35]. The higher prevalence rate provides further evidence for the clinical importance of Cubital Tunnel Syndrome (CuTS) and the need for better identification of specific risk groups [8]. In one large epidemiological study focusing on computer work as a risk factor for ulnar neuropathy, the authors found that leaning on one’s elbow while working may increase the risk of ulnar neuropathy, but not ulnar neuropathy-like symptoms [34]. In previous studies, the existence of concomitant median and ulnar nerve symptoms was observed in a large percentage of subjects, indicating the possibility of a pathophysiological predisposition or symptomatic overlap among affected individuals [8]. Patients with CTS often report symptoms outside the median nerve distribution, and previous studies indicating resolution of symptoms in the ulnar nerve distribution after carpal tunnel release [9]. Between one fifth and one third of patients with CTS also present with concomitant ulnar nerve involvement in Guyon’s canal. The pathological changes affecting the ulnar nerve are influenced by the severity of CTS. In CTS, elevated pressure within the carpal tunnel may lead to anatomical alterations of the ulnar nerve, subsequently resulting in functional impairment [36].

## 4. Pathophysiological Mechanisms of Compressive Neuropathies of the Upper Limbs

**Figure 2:**
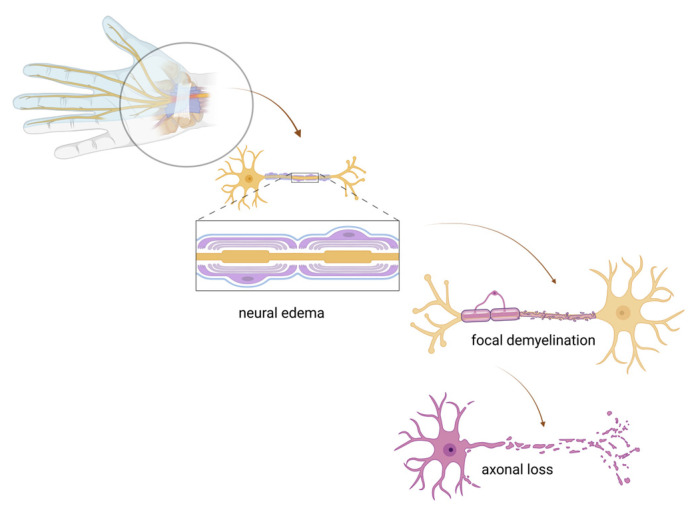
Pathophysiological mechanisms of compressive neuropathies (created in BioRender. Vulpoi, A. (2025) https://BioRender.com/io3nuo6, accessed on 17 May 2025).

Compressive neuropathies are directly linked to activities and repetitive actions at the workplace. Multifactorial etiology, repeated microtrauma and prolonged exposure time, make work related compressive neuropathies of the upper limb difficult to identify and prevent [3]. The pathophysiology of chronic compressive neuropathies of the upper limbs is attributed to repeated or prolonged compressions. In most cases, nerve compression occurs in osseocartilaginous tunnels and fascial openings. Compression may lead to microvascular damage to the nerves and their myelin sheaths. Pressure, duration, and dynamic nature influence nerve damage. Pressures below 30 mmHg cause venous occlusion, while pressures above 50 mmHg disrupt arterial blood supply, and pressures above 80 cause acute ischemia. Symptoms of intraneural microcirculation impairment may persist after an acute nerve compression [28,37]. Prolonged low-grade compression induces neural edema and focal demyelination, which are hallmarks of compression neuropathies (Figure 2). Secondarily, segmental block or slowing of nerve conduction occurs. Persistent compression may eventually lead to axonal loss [3]. Neuroinflammation plays a role in generating and maintaining entrapment neuropathy. As a result of this neuroinflammation, injured neuronal tissue becomes sensitized, contributing to the initiation and maintenance of chronic pain [3].

Chronic entrapment injuries like CTS are primarily mediated by the Schwann cell response, where decreases in internodal length and myelin thickness disrupt the efficiency of impulse propagation [38]. Disruption of the myelin sheath increases the distance between the nodes of Ranvier and interferes with impulse transmission, resulting in a decrease in conduction velocity. During an electrodiagnosis (EDX) studies, this is the first modification that has been identified [28]. Some studies have revealed that Schwann cells undergo concurrent proliferation and apoptosis after a chronic nerve injury that is independent of axonal pathology. Schwann cells proliferate rapidly when exposed to shear stress induced by laminar fluid flow. This may explain the response observed in entrapment neuropathies [39]. 

According to limited human studies, chronic compression results in marked myelin thinning of myelinated fibers. In the unmyelinated fiber population, a shift in the fiber histogram was observed due to an increased population of very small fibers. This suggests degeneration with subsequent regeneration of this fiber population [40]. Slowing of nerve conduction without axonal loss can be attributed to altered remyelination by Schwann cells after demyelination. This morphological change is characterized by a thinner myelin layer, a shorter internodal distance, and increased Schmidt-Lanterman notches along the axon compared to normal nerves [41]. Chronic nerve injury models in animals show Schwann cell proliferation at both the site of compression and at distal nerve segments. Chronic nerve compression induces Schwann cell turnover with minimal axonal injury and supports the idea that mechanical stimuli have a direct mitogenic effect on Schwann cells [42].

Repetitive movements generate friction, potentially leading to inflammation. Nerve compression can disrupt the blood-nerve barrier, impair vascular perfusion, cause edema, and result in nerve damage [28]. Persistent compression initially causes demyelination, followed by axonal degeneration. Schwann cells play a key role in both nerve injury and regeneration. Additionally, evolving fibrosis may exacerbate the effects of mechanical compression [37].

Following nerve injury, central neuronal changes are invariably involved, although the extent depends on the severity of the injury. Central sensitization, neuroinflammation, and altered cortical representations are among the changes. In patients with nerve injuries, distant neuroinflammation explains the spread of symptoms beyond dermatomes or innervation territories. Peripheral neuropathy, even mild entrapment such as CTS, may induce changes in functional and structural neuroplasticity in the primary somatosensory cortex of the brain [43]. Studies have shown that surgical decompression of the median nerve at the wrist induces changes in the postcentral gyrus, which is the main area of tactile sensory representation. In one study, changes in neuroplasticity were correlated with improvement in postoperative symptoms. This revealed a potential link between clinical outcome and functional brain reorganization [44,45].

## 5. Risk Factors Associated with Computer Use—What Do the Studies Show?

Using an input device such a keyboard or mouse involves some non-neutral postures of the hand, rendering computer users more susceptible to developing CTS. These biomechanical stressors—namely repetitive motion, prolonged wrist extension during keyboard use, and sustained forearm pronation—are key contributing factors to neuropathic symptoms observed in individuals with prolonged computer exposure [28,46]. The CTS symptoms of office workers were associated with occupational characteristics (daily work hours) as well as individual factors [47]. Subjects with over 8 years of computer work and over 12 h of work per day were at higher risk of developing CTS [4]. In another study, respondents who had worked for 5 years or longer were 7.9 times at higher risk of CTS than those who had worked less than 5 years [26].

A significant portion of the literature fails to differentiate between CTS and MNW, despite its clinical significance. MNW refers to objectively confirmed dysfunction of the median nerve at the level of the transverse carpal ligament, whereas CTS encompasses a constellation of symptoms and signs that may be present even without measurable median nerve impairment [5]. Tingling, numbness, paresthesia, and hand pain found in computer users who work many hours a day at their computers is a red flag for CTS development [28]. In recent years, there has been increasing scientific evidence that the development of CTS is promoted by highly repetitive manual tasks involving awkward hand/wrist postures, with hand flexion and extension, heavy strain, or hand/arm vibration during work. This shift is attributed to its increasing prevalence among professionals in various industries, from computer work to assembly line operations [31].

**Figure 3:**
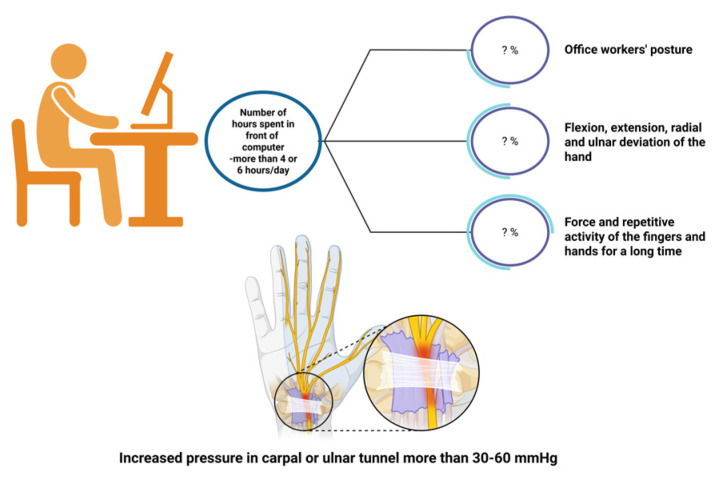
Possible causes of increased intracranial pressure among computer workers (created in BioRender. Vulpoi, A. (2025) https://BioRender.com/5phkloc, accessed on 17 May 2025).

In a 1-year follow-up study, tingling and numbness in the right hand were associated with mouse usage for more than 20 h per week, but not with using keyboard usage [48]. The wrist extension position was adopted by over 85% of people who used traditional mice and 63% of people who used flat mice [49]. A choice of any type of mouse (flat, traditional, vertical, respectively) may increase the risk of CTS, but there is no significant difference in risk between these three types of mice. Using a mouse may increase pressure on CTS patients [50]. According to an experimental study conducted by Keir P et al., the mean carpal tunnel pressure increased from 5 mmHg while resting to 17–19 mmHg after placing the hand on the mouse, and 29–33 mmHg while dragging the mouse [51]. The level of pressure that interferes with normal nerve function is 30 mm Hg. Tissue compression at 30 mm Hg causes mild neurophysiological changes and paresthesia symptoms in the hands. Compression at 60 and 90 mm Hg induced a rapid and complete blockage of sensory transmission. There is a critical pressure level between 30 and 60 mm Hg where nerve fiber viability is compromised [50]. Figure 3 illustrates the possible causes of increased pressure in the carpal tunnel among those who work with a computer mouse and keyboard.

When typing, the wrists make repetitive movements for a long time. This usually requires a mixture of force and repetitive activity of the fingers and hands for a long time. Flexed or extended hand position had higher risk for CTS [4]. However, extension, radial and ulnar deviation were more prevalent among all users. In response to a keyboarding task, the median nerve showed acute changes, and those changes were dose related. The median nerve was found to be swollen following an hour of keyboard use on ultrasound [52].

Similar to how repetitive wrist movements can lead to median neuropathy, repetitive elbow flexion and extension may result in UNE [37]. Also, external compression on the nerve is another cause of neuropathy [7]. In a study conducted on patients with UNE, intraneural pressures measured between the ulnar nerve and the overlying fibrous arcade exceeded 200 mm Hg during elbow flexion or isometric contraction of the flexor carpi ulnaris muscle. At rest, with the elbow in extension, pressures ranged between 0 and 19 mm Hg, indicating a marked increase under dynamic or stress conditions [53]. In studies conducted among computer users, both Ganeriwal et al. and Bamac et al. reported a reduction in nerve conduction velocity of the ulnar nerve at the wrist [28,46]. A plausible anatomical explanation is that the transverse carpal ligament spans Guyon’s canal, acting as a floor on the ulnar side of the hand and wrist, before transitioning seamlessly into its position as the ‘roof’ of the carpal tunnel [54]. Wrist extension, ulnar deviation during keyboarding, and mouse use are non-neutral wrist positions that can exert pressure on the hypothenar eminence, leading to ulnar nerve irritation. Moreover, resting the elbow on a hard surface during computer use may contribute to the development of ulnar neuropathy by exerting compressive forces on the nerve at the olecranon level [2].

The posture commonly adopted by office workers during computer use may contribute to the development of double crush syndrome DCS. This is translated by forward head posture, which involves lower cervical flexion, scapular abduction, and thoracic kyphosis, leading to abnormal muscle imbalance that can result in compression of the lower cervical nerve roots. The occurrence of DCS is strongly associated with entrapment of peripheral nerves: proximal compression of axon continuity leads to distal injury along the same neuronal pathways [2].

Computer use and CTS have been associated with contrasting results in the literature. Among the new version of the American Academy of Orthopedic Surgeons (AAOS) in Management of Carpal Tunnel Syndrome Evidence-Based Clinical Practice Guideline, only one low-quality study met the inclusion criteria and found an association between intensive keyboard use and CTS that was statistically significant [55]. In the previous guidelines, moderate evidence was provided supporting a link between computer work and CTS [56]. However, that guideline was based on three moderate-quality studies which were later considered low quality for the current version [55].

Several studies have identified objective neurophysiological changes associated with repetitive hand activity and sustained wrist posture [32,33]. For instance, a study by Bamac et al., found a reduced sensory nerve conduction velocity (SNCV) in the median nerve across the carpal tunnel in the hands of the computer’s users compared to the control. The dominant hand of computer users had a significantly greater delay than their non-dominant hand in SNCV [46]. One possible explanation for this could be the long-term use of a computer mouse with the dominant hand. Notably, these alterations were present in both symptomatic and asymptomatic individuals, suggesting subclinical CTS [46]. Ganeriwal et al., in their study, identify modification in sensory and motor nerve conduction for median and ulnar nerves in the wrist in computer users, who use mouse and keyboard for more than six hours a day [28]. Following one hour of continuous mouse and keyboard use, both the median and ulnar nerves exhibit alterations in cross-sectional area and neurophysiological parameters [2,52].

In conclusion, some studies have shown that intensive computer use and working without breaks are associated with an increased risk of wrist and hand symptoms by approximately 22% and 15%, respectively [1]. These employees are at risk for CTS and nerve damage caused by repetitive movements, wrist flexion and extension and non-neutral wrist postures [28,46]. They must perform a detailed clinical and neurological evaluation, including NCS [28].

## 6. Others Risk Factors and Compressive Neuropathies

It is generally understood that compressive neuropathies are not caused by a single factor. The causes of CTS can be local (e.g., cysts, tumors) [57], regional (e.g., rheumatoid arthritis) [58,59] or systemic (e.g., diabetes) [60,61]. Also, the etiology of CTS may involve occupational factors or genetic predisposition, with a higher prevalence observed in females and a peak incidence occurring between the ages of 45 and 64 [62]. In addition to physical and biomechanical factors, individual factors also play a role in determining their installation. An underlying subclinical neuropathy may indicate an increased susceptibility to developing CTS [63,64].

**Figure 4:**
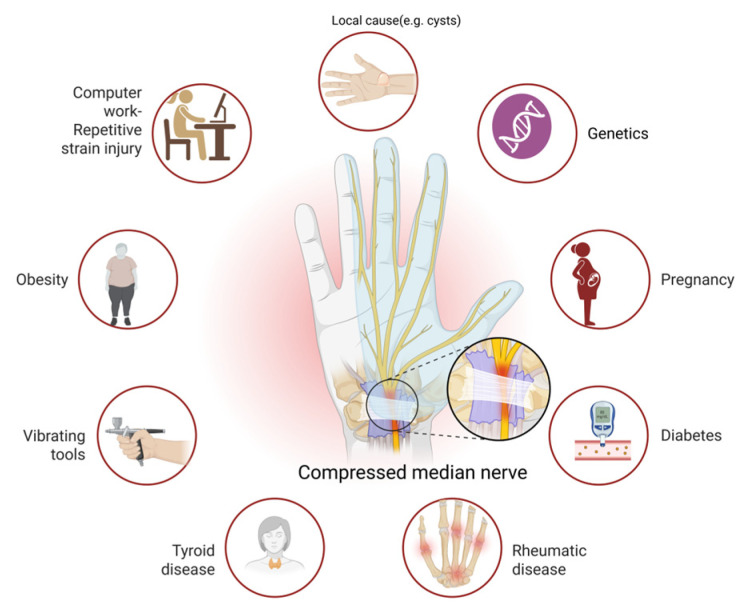
An overview of the underlying causes of carpal tunnel syndrome (created in BioRender. Vulpoi, A. (2025) https://BioRender.com/naj1m5c, accessed on 17 May 2025).

A study by Lewańska M and collaborators indicates that there are usually other causes for CTS besides working at a computer [65]. This draws attention to the relationship between CTS and potential etiological factors. In view of this, it is important to recognize that CTS can be associated with a variety of etiological factors, including obesity, diabetes mellitus, thyroid disease, rheumatic disease and vibrating tools, hormonal replacement therapy or oral contraceptives, recent menopause or tenosynovitis [65,66]. Figure 4 illustrates an overview of the underlying causes of CTS.

**Genetics**: Entrapment neuropathies have recently been associated with genetic predispositions [67]. Many of the genes are related to connective tissue and extracellular matrix architecture. It remains unclear at present whether these genes increase vulnerability by altering the nerve itself or the environment through which the nerve travels [67]. There is a genetic component to CTS, arising from altered development and growth of the musculoskeletal structure and/or abnormal connective tissue architecture. Accordingly, height is inversely correlated with CTS etiology [67]. Short stature, shorthand length, increased palm width, and a larger wrist index (wrist depth/width) are strongly linked to CTS [68].

**Gender**: According to past studies, female gender is also an independent risk factor for CTS, and men tend to have more severe symptoms of the disorder [69].

**Pregnancy**: The incidence of CTS is high during the third trimester of pregnancy (24.6%) [70]. Many cases are mild and would not disrupt functioning, so it is frequently underreported [70].

**Thyroid disease**: CTS is strongly associated with autoimmune thyroid disease, while hypothyroidism is often considered both a cause and comorbidity of the disorder [71]. Nervous system function is significantly influenced by thyroid hormones. Various neuropathies may be associated with abnormal thyroid hormone levels. The potential mechanism of nerve damage in hypothyroidism involves fluid retention, which may result in tissue swelling and subsequent compression of peripheral nerves. Also, CTS in hypothyroidism can be attributable to mucinous material or mucopolysaccharides deposited on the median nerve. An untreated hypothyroid state can also lead to swelling of the synovial membrane around the carpal tunnel tendons [72]. Hypothyroidism affects the severity of CTS symptoms and the outcome of carpal tunnel surgery [73].

**Diabetes**: Diabetes patients often suffer from musculoskeletal disorders. Studies have shown a high correlation between such complications and diabetes, but the pathophysiological reasons are largely unknown [61]. There is a highe prevalence of CTS in people with diabetes, especially those with coexisting diabetic polyneuropathy and/or long-term of the disease [74]. If diabetic polyneuropathy is present, the median nerve is more susceptible to carpal tunnel pressure [75].

**Obesity**: The risk of CTS increases significantly with an increase in body mass index (BMI) > 30 [76]. CTS related to being overweight is expected to increase as the prevalence of being overweight and obesity increases worldwide [77]. There is a correlation between low BMI and UNE and high BMI with CTS [78]. There are, however, studies which have found no correlation between high BMI and CTS and lipid profile, either based on Boston Carpal Tunnel Syndrome Questionnaire (BCTQ) or EDX results [64].

**Smoking**: According to a meta-analysis conducted by Lampainen K et al., there is no association between smoking and CTS in case–control and cohort studies [79]

## 7. Diagnostic Considerations for Median and Ulnar Neuropathy in Computer Users

Traditionally, compressive neuropathies are diagnosed using clinical history, physical exam, and electrophysiological studies. Typical symptoms of CTS include numbness, tingling, nocturnal paresthesia, and/or tenderness in the radial 3.5 fingers [62]. In the clinical setting, CuTS patients present with pain, numbness, paresthesia, and the fifth digit and medial aspects of the fourth digit [80]. These symptoms can often be exacerbated by elbow flexion, especially while sleeping. When the median or ulnar neuropathy is more severe, patients may complain of hand weakness, frequent clumsiness when grasping an object with their hands and frequent object drops [62,80].

Provocative clinical tests (Tine’s, Phalen’s test) have been shown to have low diagnostic accuracy, and the value of clinical examination has been shown to be highly variable for compressive neuropathies of the upper extremities [81,82].

NCS are currently the best way to document the severity of MNW or UNE and contribute to CTS or CuTS diagnosis. Using NCS, you can evaluate thick myelinated sensory and motor fibers, which may be useful in the workup of CTS for three reasons: to improve diagnostic accuracy for CTS and assist in the diagnosis of disorders that may mimic CTS. Additionally, it is crucial to assess the severity of CTS, which is necessary for a more appropriate treatment plan, and it serves as a baseline that can be followed over time, especially when patients have persistent or new complaints following surgery for CTS [83]. The minimum requirements for the EDX evaluation of CTS outlined in the AANEM Practice Parameters and in the Clinical Quality Measures published by the Carpal Tunnel Quality Group are testing of median sensory latency, testing of median distal motor latency, another sensory and motor nerve study in the same extremity, and, if these results are normal, followed by comparison short segment studies [84,85]. Needle EMG is not obligatory and is performed when indicated for differential diagnosis or lesion localization and may add valuable information to certain patients [5,83]. For example, EMG can be very helpful in the diagnosis of potentially mimicking disorders of CTS (cervical radiculopathies, especially C6-C7) [83]. For UNE, the most widely used EDX method is the measurement of motor conduction velocity at the elbow over a segment of 10 cm. This method is described as the ‘first line’ method in the most widely followed guidelines on the use of EDX parameters to diagnose UNE. Also, the amplitude of Compound Muscle Action Potential (CMAP) is an accurate indicator of axonal loss and therefore represents the severity of the disease. The most accurate method for assessing the severity of UNE remains EDX [86].

Another important tool for compressive neuropathies diagnosis is Neuromuscular Ultrasound (NMUS). It is increasingly used as a complement to NCS for diagnosing neuromuscular disorders and it was recently reported that some patients with clinical CTS and normal NCS may have abnormal ultrasound findings [87,88]. When diagnosing CTS, measurement of median nerve cross-sectional area at the wrist, with NMUS, may be helpful. It should also be considered in screening for structural abnormalities at the wrist in those with CTS [89]. It can be useful in patients with clinically suspected CTS who have non-localized or normal EDX, atypical EDX, failed CTS surgery, polyneuropathy, or CTS suspected to be secondary to structural pathology [90]. Also, clinicians should offer ultrasonographic measurement of the ulnar nerve cross-sectional area or diameter to confirm the diagnosis and locate the site of compression for patients with symptoms and signs suggestive of ulnar neuropathy [91]. The use of NMUS may also allow for better identification of patients with early stages of UNE who do not show any EDX patterns [92]. The addition of the second method increases diagnostic yields in mild and very mild UNEs [86].

In most cases, patients with suspected CTS can be diagnosed through clinical examination and EDX studies. However, a wrist Magnetic Resonance Imaging (MRI) is recommended in cases of uncertain clinical symptoms. This is along with those who belong to a younger age group, are of the male gender, suffer from unilateral complaints, have recurrent symptoms, or lack any pronounced predisposing factors [93].

EDX studies serve as essential tools for accurately localizing nerve injuries and characterizing their underlying pathophysiology—whether demyelination, axonal degeneration, or a combination thereof. The results provide valuable prognostic insight and inform appropriate therapeutic strategies [37]. But, combining EDX and NMUS together is more informative than using each modality separately [86,90].

## 8. Computer Work-Related Management and Prevention Recommendations

Musculoskeletal disorders are strongly influenced by the workplace environment and its components [94]. UNE, UNW and MNW appear to be one of spectrum of WMSDs affecting computer workers [32,33]. According to a systematic review, workplace strategies can include ergonomics, education, exercise, organization, physiotherapy, and epidemiological surveillance [95]. According to the studies, more promising results are obtained when interventions are combined and implemented at different levels; single-focused interventions show more limited and mixed results [95]. A scoping review from the twenty-first century found that ergonomic equipment was the most prevalent intervention strategy to prevent upper limb repetitive strain injuries amongst computer users and breaks and rest periods were deemed to be the least used intervention [96]. Taking into account the findings of the review, the following ergonomic measures are among the most recommended: using a wrist support pad to help prevent CTS, choosing a corner workstation that provides adequate forearm support, and selecting a computer mouse that promotes a neutral forearm position and minimizes pronation, which may help protect the ulnar nerve at the wrist. Additionally, when using a keyboard, it is advisable to type with a light touch, maintain the wrists in a neutral position, and consider using a split keyboard or a wrist rest for added ergonomic support [96]. The use of specialized keyboards and mice positions the user’s shoulders and forearms more neutrally, thereby reducing the number of ulnar deviations of the hand, preventing gravity from flexing the wrist, and reducing direct pressure on the carpal tunnel [97]. However, ergonomic optimization is dependent not only on equipment but also on education and behavior modification. It should be remembered that very few prospective randomized studies have been conducted on the use of neutralizing and positioning devices [97,98,99]. Rest breaks of 5 and 10 min after every hour of work have favorably affected discomfort or complaints, as well as incorporating 30 s microbreaks at 20 min intervals. Wrist exercises, like moving the wrist in a circular direction at regular intervals, are recommended for the prevention of CTS [96]. Computer users with upper limb problems, such as CTS, may benefit from carefully chosen devices. However, not enough high-quality data is available to guide decision-making in this area. Education and behavior modification are equally important or even more so than the use of special computer devices [97]. In one study among office workers, regular prevention exercise (3 times/week) was found to increase forearm muscle strength. Regular exercise improved hand grip strength and pincer grip strength in office workers with CTS symptoms. Two months of exercise improves functional status but does not affect pain complaints [100]. The optimal workplace setup requires that all major joints be kept in a neutral position. The computer monitor should be placed at a comfortable distance, allowing the user to read text easily while maintaining an upright head and trunk posture, with the back properly supported by the chair. Generally, between 20 and 40 inches (Arm’s length) from the eye to the front surface of the computer screen is considered as an ideal viewing distance. For optimal ergonomics when working on a computer, the hands should be free to float over the keyboard and the elbows and wrists should be supported. Mouse should be at the same height as the keyboard, to either side of it. The position the mouse should allow the user to maintain a straight, neutral wrist posture. In addition, it is recommended that the seat back angle be 90 degrees, with the feet on the floor or on a footrest for individuals with short legs. Additionally, the seat height and the lumbar support can be adjusted [97].

According to the study by Ekinci et al., ergonomic training can be used to prevent musculoskeletal problems in the long run. Ergonomic training provided to office workers had a positive impact on workplace awareness in both the immediate and long-term assessments [101].

## 9. Discussions

Numerous studies have shown that individuals who work extensively with computers are at increased risk for upper extremity musculoskeletal disorders, particularly CTS. Additionally, evidence suggests a relationship between ulnar nerve sensory abnormalities, functional disability, and known CTS risk factors. These findings underscore the not-uncommon involvement of the ulnar nerve in the clinical presentation of CTS [102]. Repetitive mechanical stress on the elbows or wrists during prolonged keyboard or mouse use has been associated with the development of UNE and Guyon’s canal syndrome, indicating that sustained computer work may serve as a contributing etiological factor in these neuropathies [34,46]. Despite the extensive literature on the topic, the association between computer use and CTS remains inconclusive, primarily due to limitations in the available data. As a foundation for our analysis, we have drawn upon two pivotal high-level studies: Thomsen et al. (2008) and Shiri et al. (2015) [18,19]. The findings indicate that the epidemiological evidence linking computer use to CTS remains inconclusive. Nevertheless, Shiri et al. suggest that excessive computer use, particularly prolonged mouse use—may represent a minor occupational risk factor for the development of CTS [19]. In studies conducted over the past decade that relied solely on self-reported symptoms, clinical examination maneuvers, and the Boston Carpal Tunnel Questionnaire (BCTQ), the reported prevalence of carpal tunnel syndrome (CTS) among computer users has varied significantly, ranging from 9.6% to 64% [1,25,26,27]. This wide range likely reflects inconsistencies in diagnostic criteria and methodologies across studies. In contrast, Mediouni et al. (2015) reported a considerably lower prevalence of CTS—2.3% and 4.3%, respectively [22]. However, in their longitudinal observational study based on the Cosali cohort, NCS were not employed, which may have compromised diagnostic accuracy and led to an underestimation of true CTS prevalence. These discrepancies underscore the critical role of diagnostic methods in determining the reported prevalence of CTS across studies [22]. The choice of diagnostic tools significantly influences the reported prevalence of CTS. Studies relying solely on self-reported questionnaires, structured interviews, and physician-based clinical diagnoses—without the use of objective confirmatory methods such as NCS—may overestimate the incidence of CTS, particularly among computer users. This limitation was underscored in a study involving individuals performing repetitive and forceful hand movements, where the authors emphasized that the absence of standardized diagnostic criteria and electrophysiological confirmation could lead to inflated prevalence rates [103]. Further supporting this concern, a study involving individuals with a prior diagnosis of CTS who used computers for at least one hour per day demonstrated that occupational exposure to computer work exacerbates both subjective symptoms and objective clinical findings of CTS. Moreover, these occupational factors were found to contribute to both temporary and permanent work incapacity.

From a pathophysiological standpoint, repetitive wrist movements—such as flexion, extension, supination, and pronation associated with mouse use—are recognized risk factors for CTS. In parallel, sustained elbow flexion at angles below 90 degrees during prolonged keyboard use has been shown to predispose individuals to CuTS. In both cases, nerve injury is primarily caused by excessive intracanal pressure at the wrist and elbow, leading to focal chronic ischemia. Persistent elevation of pressure results in demyelination and reduced nerve conduction velocities, as demonstrated in EDX studies.

Prolonged computer use in inadequate postures may contribute to cervical disk pathology, with proximal nerve root compression potentially impairing axoplasmic flow and predisposing peripheral nerves to secondary entrapment. This mechanism supports the concept of DCS, wherein proximal nerve compromise increases susceptibility to distal compressive neuropathies, such as median nerve entrapment at the wrist and ulnar nerve entrapment at the elbow or wrist.

For this reason, CTS prevention programs should be implemented among office workers since they can limit the functional capacity of the hand and the ability to work.

A comfortable and ergonomically optimized work environment is crucial for computer users in contemporary occupational settings. The application of ergonomic principles plays a key role in minimizing the risk of developing computer-related musculoskeletal disorders. Nevertheless, awareness and understanding of ergonomics remain limited among many computer users. There is often insufficient knowledge about the effects of cumulative trauma, the goals of ergonomic interventions, and the early signs and risk factors associated with musculoskeletal conditions, which hinders the effective implementation of preventive measures [104].

Computer users with predisposing risk factors—such as diabetes, hypothyroidism, or rheumatologic conditions—are likely at increased risk for developing compressive neuropathies. Tingling, numbness, paresthesia, and hand pain in individuals with prolonged daily computer use may raise suspicion for CTS or CuTS. However, diagnosis must be confirmed through paraclinical investigations. A symptom-based diagnosis, even when strongly suggestive from anamnestic and clinical perspectives, can be misleading. Cervical radiculopathies may mimic compressive peripheral neuropathies. From the perspective of DCS, which may occur in individuals using computers at work due to inadequate posture and repetitive movements, it is essential that, in addition to history taking and neurological clinical examination, paraclinical investigations such as EDX studies and NMUS be included as part of the diagnostic process. Additionally, incorporating NMUS during the diagnostic process can help identify structural causes of nerve compression, thereby enhancing diagnostic accuracy and guiding clinical decision-making.

Although computer use has been widely studied, our narrative review did not establish it as a definitive risk factor for compressive neuropathies. However, the evidence does support a significant association, particularly in the presence of contributing factors.

## 10. Future Directions and Conclusions

Although extensively investigated, the association between computer use and the development of compressive neuropathies—most notably CTS and CuTS—remains inconclusive. Numerous studies have reported an increased prevalence of symptoms and subclinical abnormalities among long-term computer users, particularly in the context of sustained non-neutral wrist and elbow positions, repetitive upper limb movements, and prolonged interaction with input devices. Nevertheless, a definitive causal relationship has yet to be unequivocally established.

Future studies should focus on longitudinal, multicenter prospective designs with standardized diagnostic criteria, objective assessments (such as NCS), and adequate control of confounding factors (e.g., metabolic conditions, ergonomic environment, and occupational intensity). The combined use of electrophysiological assessments and imaging modalities holds promise for enhancing diagnostic accuracy and identifying compressive neuropathies that may not be evident through clinical evaluation alone.

Moreover, clinicians must have access to evidence-based guidelines for evaluating work-related nerve dysfunctions. It would be helpful if office workers at risk were screened for compressive neuropathies on a standardized basis.

However, preventive strategies remain crucial. Workplace ergonomics, regular breaks, posture correction, and employee education should be emphasized across industries involving prolonged computer use.

It has been reported that, in addition to long-term strain on the hands and wrists in an awkward position, repetition, and strong hand/arm effort, some non-occupational risks may also contribute to CTS development, including genetics, female gender, age, obesity, diabetes, thyroid diseases, inflammatory arthritis, hand trauma, and pregnancy. Calculating risk scores would be beneficial in such cases, as it could enable the implementation of personalized management strategies—preventive, diagnostic, and therapeutic—based on the identified level of risk. In individuals with certain susceptibilities, professional activity may act as a trigger for compressive neuropathies. Such risk scores could facilitate the identification of these individuals and enable more rapid intervention.

In conclusion, while computer use has not been definitively established as a direct cause of upper limb compressive neuropathies, it remains a contributing factor within a multifactorial framework of risks. Office workers are at high risk of CTS and CuTS, which have a negative impact on their function and quality of life. Advances in research, diagnostics, and prioritization of preventative measures are essential to addressing this evolving occupational health issue.

## Figures and Tables

**Table 1:** Meta-analyses and systematic reviews of the association between computer use and CTS in the last twenty years.

First Author/Ref.	Type of Study	Sample Size	Conclusions
Thomsen J.F et al. (2008) [18]	Systematic review	8 studies	-Epidemiological data on the link between computer use and CTS remain inconclusive, as no definitive study has yet clarified the relationship
Shiri R et al. (2014) [19]	Meta-analysis	12 studies	-Excessive computer use, particularly prolonged mouse use, may be a minor occupational risk factor for CTS. Further prospective studies in office workers are needed, using objective measures of device use and CTS confirmation via NCS.
Mediouni Z et al. (2014) [20]	Meta-analysis	6 studies	-No overall link was found between computer use and CTS, though specific work conditions may still pose an increased risk.
Bibi M et al. (2019) [21]	Systematic review	8 studies	Prolonged keyboard and mouse use are considered key contributing factors in the development of CTS.

**Table 2:** Studies to the association between computer use and CTS in the last ten years.

First Author/Ref.	Type of Study	Sample Size	Prevalence of CTS (%)	Confirmed with NCS
Mediouni Z et al. (2015) [22]	Observational longitudinal cohort study (3 years)	1551 (Cosali study) 711 (PrediCTS study)	2.3% 4.3%	No Yes
Bhanderi J et al. (2017) [23]	Case–control study (1.5 years)	411 (137 cases and 274 controls)	-	No
Bibi M et al. (2019) [25]	Cross-sectional study	247	15.5%	No
Feng B et al. (2021) [1]	Cross-sectional study	969	9.6%	No
Demissie B et al. (2023) [26]	Cross-sectional study	422	11.7%	No
Kurane et al. (2025) [27]	Cross-sectional study	150	64.54%	No

**Table 3:** Computer work and ulnar neuropathies studies.

First Author/Ref.	Type of Study	Sample Size	Confirmed with NCS	Conclusions
Conlon FC et al. (2005) [32]	Cross-sectional study (1 year)	202	yes	-13.9% prevalance of upper extremity entrapment neuropaty at the wrist; -Prevalence of UNW 1.8% (right), 2.9% (left). -Prevalance of CTS 10.3% (right), 3.4% (left); -The risk of entrapment neuropathy increased with increasing hours of computer use per week.
Nainzadeh NK et al. (2011) [33]	Case series (4 years)	148	yes	-Prevalance of UNE was identified 74.5% (105); -Of the 105 with UNE, 41 had co-existing CTS;
Andersen JH et al. (2012) [34]	Case-referent study (6 years)	546 UNE	yes	-A negative exposure-response relation between hours of daily computer use and ulnar neuropathy;

## Data Availability

The data are available in the cited sources.

## Abbreviations

The following abbreviations are used in this manuscript:

AAOS	American Academy of Orthopedic Surgeons
ACGIH	American Conference of Governmental Industrial Hygienists
BCTQ	Boston Carpal Tunnel Syndrome Questionnaire
BMI	Body Mass Index
CTS	Carpal Tunnel Syndrome
CMAP	Compound Muscle Action Potential
CuTS	Cubital Tunnel Syndrome
DCS	Double Crush Syndrome
EDX	Electrodiagnosis
HAL	Hand-Activity Level
MNW	Median Neuropathy at the Wrist
MRI	Magnetic Resonance Imaging
NCS	Nerve Conduction Studies
NMUS	Neuromuscular Ultrasound
TLV	Threshold Limit Value
UNE	Ulnar Neuropathy at the Elbow
UNW	Ulnar Neuropathy at the Wrist
WMSDs	Work-related Musculoskeletal Disorders
